# Comparison of admitting neutrophil/lymphocyte ratio with baseline NIH stroke scale score in discriminating poor 30-day stroke outcome among Nigerian Africans

**DOI:** 10.3389/fstro.2025.1562048

**Published:** 2025-04-09

**Authors:** Oladotun V. Olalusi, Joseph Yaria, Akintomiwa Makanjuola, Rufus Akinyemi, Mayowa Owolabi, Adesola Ogunniyi

**Affiliations:** 1 Department of Neurology, University College Hospital, Ibadan, Nigeria; 2 Neuroscience and Aging Research Unit, Institute of Advanced Medical Research and Training, College of Medicine, University of Ibadan, Ibadan, Nigeria; 3 Center for Genomics and Precision Medicine, College of Medicine, University of Ibadan, Ibadan, Nigeria; 4 College of Medicine, University of Ibadan, Ibadan, Nigeria; 5 Department of Medicine, Lebanese American University of Beirut, Beirut, Lebanon; 6 Department of Medicine, Blossom Specialist Medical Center, Ibadan, Nigeria

**Keywords:** neutrophil-lymphocyte ratio (NLR), NIH Stroke Scale (NIHSS) score, modified Rankins scale (mRS) score, acute ischemic stroke (AIS), 30-day functional outcome, ischemic stroke mortality, Nigerian Africans, Indigenous West Africans

## Abstract

**Aim:**

The National Institutes of Health Stroke Scale (NIHSS) score is an established marker of stroke severity. Its use is time-consuming and requires formal training for optimal results. In contrast, the neutrophil-lymphocyte ratio (NLR), known to be independently associated with stroke outcome, can be readily calculated from routine peripheral blood counts with minimal training. We hypothesized that the NLR may perform similarly to the NIHSS score, in discriminating persons with poor 30-day stroke outcome, in a low-resource setting.

**Methods:**

We followed up 106 participants with clinico-radiologic diagnosis of first-ever acute ischemic stroke (AIS). Patients with clinico-laboratory features of fever, aspiration pneumonia, sepsis, or infection were excluded at baseline. The NLR was obtained at admission while the functional outcome was assessed using the modified Rankin scale (mRS) score at day 30. Receiver operating characteristics (ROC) curves and Cox proportional hazards were used to determine the discriminatory ability of the NLR compared with the NIHSS score in identifying patients with poor 30-day stroke outcome (mRS > 3). The respective areas under the curves (AUC) and HRs (95%CI) were documented.

**Results:**

The median interquartile range (IQR) NLR of the study population was 2.87 (3.0). Patients in the higher tertiles of NLR had higher mean standard deviation 30-day mRS scores of 4.9 (1.2) compared to the middle 3.3 (1.2) and lower tertiles 2.3 (1.2) (*p* < 0.001). Admitting NLR had an AUC (95% CI) of 0.83 (0.75–0.91) and HR (95%CI) of 1.19 (1.01–1.40) compared to admitting NIHSS score with AUC of 0.89 (0.84–0.95) and HR of 1.25 (1.14–1.37) in discriminating poor 30-day outcome.

**Conclusion:**

The NLR alone performed similarly to the NIHSS score and may help identify patients with adverse 30-day AIS outcome in low-resource settings.

## Introduction

The National Institutes of Health Stroke Scale (NIHSS), a 15-item neurologic examination scale initially developed for acute stroke research, is a well-known, reliable, and validated stroke impairment scale (Brott et al., 1989; Goldstein et al., 1989). It has shown significant reliability in diverse groups, clinical settings, and languages and is the benchmark for many acute care decisions, including thrombolytic therapy (Kasner et al., 1999). The NIHSS however is not without several drawbacks (De Haan et al., 1993; Lyden et al., 2001; Meyer and Lyden, 2009; Meyer et al., 2002). It contains items with poor reliability and redundancy, poorly accesses right hemispheric and posterior circulation stroke, poorly quantifies certain deficits (Martin-Schild et al., 2011) (e.g., deafness, hand grip), and gives no insight into the contribution of inflammatory-immune response to stroke (Makharia et al., 2024; Woo et al., 1999; Dewey et al., 1999). Additionally, the use of the NIHSS often requires specific training and certification (Goldstein and Samsa, 1997), often non-existent in many low-resource settings (Schmülling et al., 1998). In such settings, many patients with stroke are initially seen by non-neurologists who find the NIHSS complex, cumbersome, and time-consuming (Tirschwell et al., 2002; Owolabi and Platz, 2008).

In contrast to the NIHSS, the neutrophil-lymphocyte ratio (NLR) is an inflammatory biomarker that is readily calculated from routine peripheral blood count (Elkind et al., 2001; Yu et al., 2018)—a cheap and widely available laboratory investigation (Omuse et al., 2018). In many parts of Sub-Saharan Africa, the complete blood count costs <2 USD and is quick (easily obtained from automated counters) and routinely done for all in-patients as part of baseline investigations (Omuse et al., 2018; Ni, 2016). The NLR has been shown to be associated with stroke severity and outcome (Elkind et al., 2001; Yu et al., 2018), and unlike the NIHSS, gives an insight into the inflammatory burden of stroke. Inflammatory and immune response is known to be central to stroke pathobiology and outcome (Elkind et al., 2001) as inflammatory cells are involved in all the stages of acute stroke—from initial artery occlusion to brain parenchymal damage, the subsequent tissue repair, and the development of various complications (Nam et al., 2018).

The biochemical relationship between high NLR and acute stroke can be described as bidirectional. While leucocytosis (Kim et al., 2016) and high NLR have been linked to atherosclerosis, plaque instability, and rupture, leading to acute thrombotic events, other studies have also shown that an elevated white blood cell count may be associated with poor outcomes in patients with ischemic stroke (Grau et al., 2004). Central nervous system (CNS) injury, including severe stroke, induces immunodepression leading to secondary immunodeficiency in a phenomenon known as CNS injury-induced immunodepression (CIDS) (Meisel et al., 2005; Dirnagl et al., 2007; Macrez et al., 2011). Focal cerebral ischemia induces an extensive apoptotic loss of lymphocytes, a shift from T-helper cell (Th1) to Th2 cytokine production (Meisel et al., 2005), with atrophy of secondary lymphatic organs (spleen and thymus), thus increasing the risk of post-stroke complications (Offner et al., 2006), further worsening functional outcome (Zhao et al., 2016).

While the potential utility of admitting NLR in acute stroke care has been documented, albeit largely in the Global North, there is limited evidence of a head-to-head comparison with a stroke severity marker, especially in resource-limited settings. Due to the genetic deletion of the Duffy antigen receptor for chemokines (a receptor for *Plasmodium vivax* malaria), Indigenous Africans are known to have lower baseline leucocyte counts, NLR, and neutrophils levels (benign leucopenia) (Thobakgale, 2014; Reed and Diehl, 1991; Lim et al., 2010; Reich et al., 2009) compared to Caucasians. This raises curiosity for a potentially divergent contribution of NLR to stroke outcome among Africans, known to have more severe stroke (Adebayo et al., 2023). We hypothesized that the NLR, used alone, may perform similarly to the NIHSS score in discriminating persons with poor 30-day stroke outcome, in a low-resource setting, with few personnel trained to assess the NIHSS score.

## Methods

### Study design/site

This was a prospective cohort study. We studied 106 patients with first-ever acute ischemic stroke (AIS) at University College Hospital—a 1,000-bed federal tertiary hospital in Ibadan, Oyo state, southwest Nigeria. Stroke participants were followed up for 30 days. Cohort inception was January 2022, and the last patient was seen (on follow-up) in October 2022. Ethical approval was obtained from the Joint University of Ibadan/University College Hospital Institutional Review Board with approval number NHREC/05/01/2008a.

### Study population

The study population consisted of consenting patients aged 18+ years presenting with first-ever AIS, within 72 h of ictus—confirmed using neuroimaging modalities. Patients were excluded if they had: (1) clinico-laboratory evidence of infection/sepsis at admission (which may cause admitting neutrophilia and thus confound the baseline NLR); (2) hemorrhagic stroke (a more severe stroke subtype with higher risk of infectious complications at admission) (Komolafe et al., 2024); (3) previous stroke/pre-morbid mRS >0 (associated with pre-stroke functional disability); (4) underlying chronic immune/inflammatory disease, hematologic malignancy, or use of NSAIDS/steroids or immune modulatory agents.

### Sample size

The minimum sample size of the cases was calculated (Charan and Biswas, 2013) with Za^1^ set at 1.96 (95% confidence interval), power of study (Zb) set at 90% = 1.28, and standard deviation of mRS in stroke was 2.71+/−1.01 as determined in a Nigerian study (Ojagbemi et al., 2013). The proposed difference (M) in mRS between subjects with high NLR and subjects with low NLR was 0.5, and a minimum sample size (N) of ≈86 was obtained. Allowing for an attrition rate of 15%, the minimum sample size was approximated to 100.

### Sampling technique

All subjects who met the inclusion criteria were recruited consecutively until the determined sample size was reached. These patients were recruited in the emergency room and medical wards. The first patient was seen in January 16, 2022, and the last patient was seen on follow-up in October 30, 2022.

### Definition of outcomes of interest

Stroke definition: Ischemic stroke was defined, using the updated definition of stroke for the 21^st^ century proposed by the American Heart Association/American Stroke Association (AHA/ASA) (Sacco et al., 2013). All cases were evaluated clinically and the diagnosis of stroke was confirmed using neuroimaging (CT scan or MRI). Infarct size and location were measured and documented. Infarct was measured using the computerized planimetry of the neuroimaging scanners. Clinically, the infarct size was determined using the geometric ellipsoid model popularized by (Kothari et al. 1996). This method has been found to be fast and easily reproducible, and has a high inter-rater and intra-rater reliability. It also corresponds well with infarct size as measured by computerized planimetry for middle cerebral artery infarct and lacunar infarct. The formula used is: V = A × B × C/2, where: V = infarct volume, A = largest lesion diameter, B = a second line drawn perpendicular (at 90 [0]) to A at the widest dimension, and C= approximate number of CT slices with lesion multiplied by slice thickness. Volume of infarct was recorded in ml/cm (Kasner et al., 1999). Stroke location was defined as subcortical, cortical-subcortical (referred to herein as cortical), brainstem (midbrain, pons, medulla), and cerebellar. The Trial of Org 10172 in Acute Stroke Treatment (TOAST) classification was further used to characterize stroke mechanism.

The complete blood count was obtained at presentation, and the NLR was calculated as a ratio obtained by dividing the absolute neutrophil count by the absolute lymphocyte count. Patients were closely followed up during in-patient care and intra-hospital complications were documented. The outcome at 30 days was assessed using the modified Rankin scale. Participants were stratified into two outcome categories: good outcome (mRS 0–3); and poor outcome (mRS 4–6). An mRS cut-off score of 3 was used based on findings from previous studies in the region (Akinyemi et al., 2021; Sarfo et al., 2023; Obiako et al., 2011)—Africans are known to have worse stroke outcomes than Caucasians (Akinyemi et al., 2021; Sarfo et al., 2023; Obiako et al., 2011; Ekeh et al., 2015), attributable to delayed stroke recognition/presentation, poor risk factor control, limited access to diagnostic/interventional services, lack of stroke units, and payment for care out-of-pocket, among others. Patients who did not survive up to 30 days had the date of death noted; patients who were discharged before 30 days post-stroke were followed up as outpatients in the clinic and via telephone inquiry.

### Study procedure

At inception, all potential study participants were screened for infection/sepsis using infection-exclusion parameters from the Centers for Disease Control and Prevention/National Healthcare Safety Network (CDC-NHSN) surveillance definition for healthcare-associated infections (see Supplementary Table S1). A battery of clinical and laboratory evaluations (assessing for fever, infective respiratory symptoms, urinary symptoms, gastrointestinal symptoms, and ulcers on the skin) and simple bedside procedures (urinalysis and pulse oximetry) to determine the presence of urinary tract infections (UTI), pneumonia, gastroenteritis, and skin ulcers. Laboratory parameters such as complete blood count, C-reactive protein, chest radiograph, urine culture, and sputum culture were requested, and those with clinical and/or laboratory evidence of infection/sepsis were excluded. The participant recruitment flowchart is shown in Supplementary Figure S1.

Using a structured interviewer-administered questionnaire, data regarding sociodemographic characteristics, details of the index stroke including symptoms, relevant aspects of physical and neurological examination findings, disability status before stroke, presence or otherwise of vascular risk factors, and medical history were collected from each selected participant after consent. Stroke severity as assessed using the NIHSS score was recorded. Participants' neuroimaging findings (obtained from a 64-slice GE Revolution^TM^ maxima CT scanner and a 0.5T MRI scanner) and results of relevant laboratory investigations (including the full blood count obtained from a 5-count automated hematology analyzer using the principle of volume conductivity scatter) were also documented.

Participants were followed up during hospitalization. All patients received standard stroke care as per the guidelines at the Neurology Unit, University College Hospital Ibadan, which is modified from foreign guidelines to suit local realities. There was no intravenous thrombolysis (IVT) or endovascular thrombectomy (EVT) available to our patients due to prohibitive costs, lack of functional health insurance packages, late presentation, and suboptimal human/material capacity for acute interventional care. Functional outcome on the 30th day post-stroke was assessed using the modified Rankin scale. Participants who were discharged before 30 days were followed up at the neurology outpatient clinic on the 30th day post-stroke and functional outcome was assessed. To facilitate this, each participant was contacted by telephone 2 days before and a day before the clinic appointment.

### Data analysis

Study participants were classified into tertiles based on NLR on admission. To determine the relationship between baseline NLR, admitting NIHSS score, and 30-day functional outcome (mRS), bivariate analyses were performed using relevant statistical tests: chi-square (or Fisher's exact) test for categorical variables; ANOVA for normally distributed continuous variables, or its non-parametric equivalent (Mann–Whitney U/Kruskal–Wallis test) for non-normally distributed continuous data. Spearman correlations were used to test the association between baseline NLR, admitting NIHSS score, and 30-day mRS score. A receiver operating characteristics (ROC) curve was then used to determine the discriminatory ability of the NLR compared with the NIHSS score in identifying patients with poor 30-day stroke outcome (mRS > 3). Having adjusted for age, gender, presence of hypertension, presence of type 2 diabetes mellitus (T2DM), and admitting Glasgow coma score (GCS), Cox proportional regression was used to determine the predictive ability of admitting NLR compared with the NIHSS score in determining adverse 30-day functional outcome. The respective AUCs (95% CI) and hazard ratios (HR, 95 CI) were documented. A *p*-value of <0.05 was deemed statistically significant.

## Results

Table 1 shows the baseline characteristics of the study participants. The mean (SD) age of study participants was 64.38 (13.3) and 56.6% were male. The most common stroke risk factor was hypertension (88%), followed by T2DM (26%). Using the TOAST classification, 41 (38.7%) of the subjects had small vessel disease, 34 (32.1%) had stroke of undetermined etiology, 18 (17%) had cardioembolic stroke, 12 (11.3%) had large vessel disease, and 1 (0.9%) had stroke of other determined etiology. There were equal number of participants with subcortical 48 (45.3%) and cortical stroke 48 (45.3%), while six (5.7%) of the patients had brainstem stroke, and four (3.8%) had cerebellar stroke.

**Table 1:** Sociodemographic, clinical, and laboratory characteristics of study participants.

Variables	Cases (*N* = 106)
Age (years), mean (SD)	64.38 (13.3)
Gender, *N* (%)	
Male	60 (56.6)
Female	46 (43.4)
Marital status, *N* (%)	
Single	3 (2.8)
Married	100 (94.3)
Separated	3 (2.8)
Ethnicity, *N* (%)	
Hausa	5 (4.7)
Igbo	8 (7.5)
Yoruba	90 (84.9)
Others	3 (2.8)
Socioeconomic status, *N* (%)	
Low	29 (27.4)
Medium	77 (72.6)
High	0(0.0)
Household setting, *N* (%)	
Rural	28 (26.4)
Urban	78 (73.6)
Years of education, *N* (%)	
0–5 (primary)	19 (17.9)
5–10 (secondary)	29 (27.4)
>10 (tertiary)	58 (54.7)
Hypertension, *N* (%)	93 (87.7%)
Diabetes mellitus, *N* (%)	28 (26.4%)
Smoking, *N* (%)	
Never	100 (94.3%)
Ex	4 (3.8%)
Current	2 (1.9%)
Alcohol, *N* (%)	
Never	87 (82.1%)
Ex	12 (11.3%)
Current	7 (6.6%)
Waist-hip ratio (WHR), Mean (SD)	0.98 ± 0.1
Mean (SD) admitting systolic BP, mmHg	156.99 ± 27.77
Mean (SD) admitting diastolic BP, mmHgMedian (IQR) admitting NIHSS	93.04 ± 15.65 12.00 (8.25)
Mean (SD) admitting NIHSS	12.90 (5.30)
Mean (SD) admitting GCS	13.70 (2.37)
Pre-morbid mRS score, mean (SD)	0 (0)
Mean (SD) admitting mRS score	4.02 (1.70)
Time to presentation, in hours mean (SD)	30.00 (23.70)
Total WBC/μl, median (IQR)	7,625.0 (2,797.5)
Neutrophil count, % median (IQR)	66.5 (17.6)
Lymphocyte count, % median (IQR)	23.2 (15.5)
Monocyte count, %	9.2 (3.0)
Platelet count/ul	221,632.1 (83,301.2)
NLR, median (IQR)	2.87 (3.0)
PCV, %, mean (SD)	39.6(5.3)
RBG, mg/dl, mean (SD)	138.3 ± 54.8
Serum TC, mg/dl, mean (SD)	206.3 ± 62.7
Serum LDL, mg/dl, mean (SD)	133 ± 51.1
Serum HDL, mg/dl, mean (SD)	51.7 ± 17.6
Serum TG, mg/dl, mean (SD)	106.4 ± 39.8
Median (IQR) infarct size, cm	2.9 (3.95)
Median (IQR) infarct volume, mls	10.00 (25.21)
Mean (SD) 30-day mRS score	3.52 (1.60)
Median (IQR) mRS score	3.0 (3.0)
IVT/EVT, *N* (%)	0 (0)
30-day fatality *N* (%)	20 (18.9)

WBC, white blood cells; NLR, neutrophil-lymphocyte ratio; PCV, packed cell volume; RBG, random blood glucose; GCS, Glasgow coma scale score; mRS, modified Rankins scale score; NIHSS, National Institutes of Health Stroke Scale; NLR, neutrophil-lymphocyte ratio; RBG, random blood glucose; LDL, low-density lipoprotein cholesterol; HDL, high-density lipoprotein cholesterol; TC, total cholesterol; TG, triglyceride; Cr, creatinine; ED, emergency department; IVT, intravenous thrombolysis; EVT, endovascular thrombectomy.

The median (IQR) NLR and NIHSS of study participants was 2.87 (3.0) and 12.0 (8.25), respectively. The median (IQR) 30-day mRS score of study participants was 3.00 (3.0). At 30-day follow-up, a total of 20 patients (18.9%) had fatal outcome (mRS 6), 11 (10.4%) had severe functional disability (mRS 5), 16 (15.1%) had moderate-severe disability (mRS 4), while 24 (22.6%) had moderate disability (mRS 3), 27 (25.5%) had mild disability (mRS 2), and 8 (7.5%) had no disabling symptoms (mRS 1).

**Figure 1:**
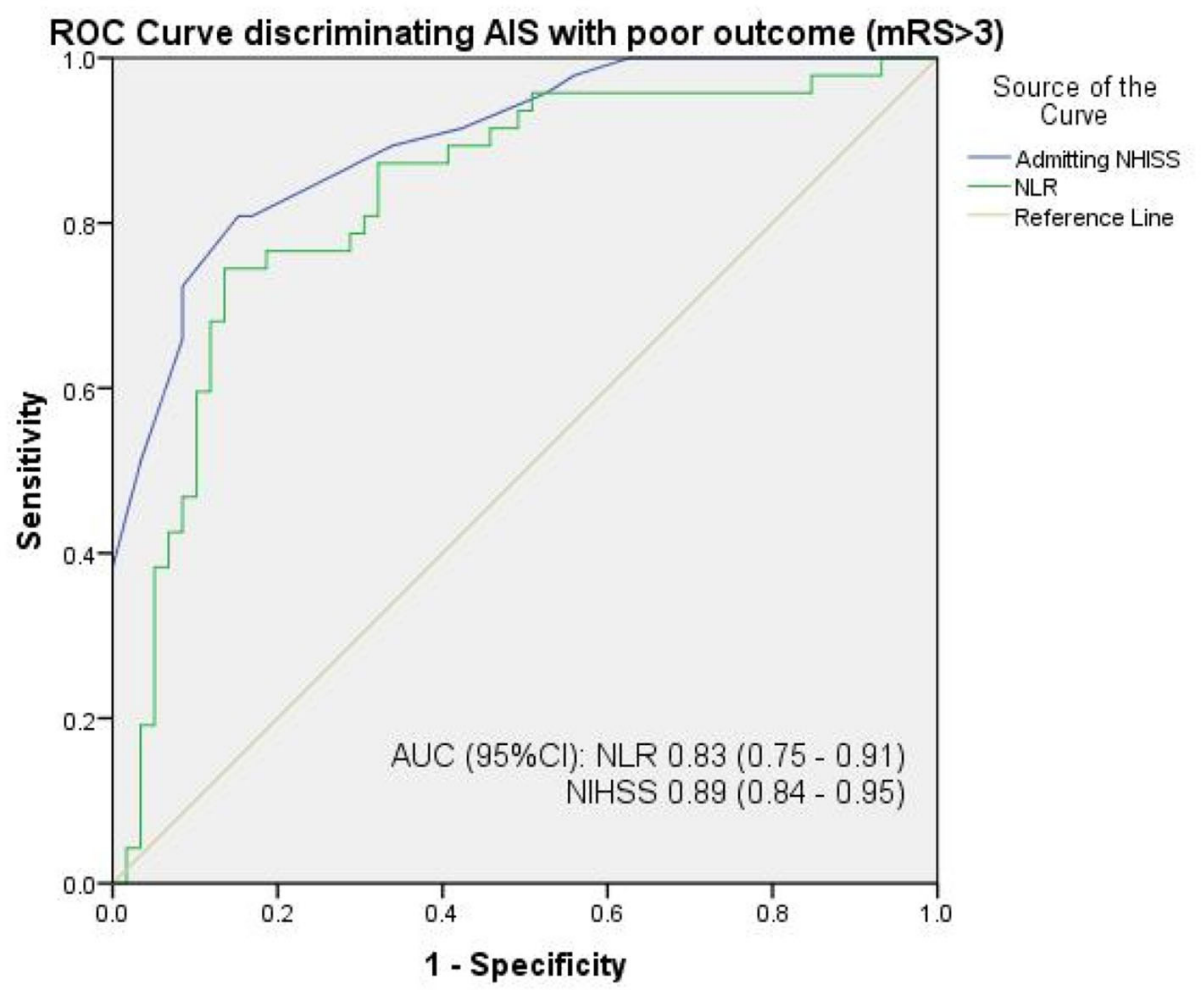
ROC curve showing the discriminating ability of the NLR compared with the NIHSS in identifying patients with adverse AIS outcome. The optimal cut-off values (with corresponding sensitivity and specificity) were 2.71 (81%, 70%) for the NLR and 12.5 (81%, 83%) for the NIHSS score, respectively. NLR, neutrophil-lymphocyte ratio; NIHSS, National Institutes of Health Stroke Scale score; AIS, acute ischemic stroke.

Table 2 shows the baseline sociodemographic, clinical, and laboratory characteristics of stroke participants according to NLR tertiles. Patients in the highest NLR tertiles had higher median (IQR) 30-day mRS scores of 5.0 (2.0) compared to the middle 3.0 (1.0) and lower tertiles 2.0 (1.0) (*p* < 0.001). Similarly, patients in the highest NLR tertile had higher mean (SD) admitting NIHSS scores of 17.6 (4.6) compared to the middle 12.5 (3.3) and lower tertiles 8.5 (3.2) (*p* < 0.001). Baseline NLR and admitting NIHSS score correlated positively with the 30-day functional outcome, with r = 0.67 (<0.001) and r = 0.79 (<0.001), respectively. In discriminating poor 30-day functional outcome, the AUC (95% CI) of the admitting NLR alone was 0.83 (0.75–0.91) compared to the NIHSS score with 0.89 (0.84–0.95) (Figure 1). The optimal cut-off values (with corresponding sensitivity and specificity) were 2.71 (81%, 70%) for the NLR and 12.5 (81%, 83%) for the NIHSS score, respectively. Having adjusted for age, gender, presence of hypertension, presence of T2DM, and admitting GCS, admitting NLR had an HR (95%CI) of 1.19 (1.01–1.40) compared to admitting NIHSS score with HR (95%CI) of 1.25 (1.14–1.37) in identifying patients with adverse 30-day outcome (Table 3).

**Table 2:** Sociodemographic, clinical, and laboratory variables of stroke participants according to NLR tertiles.

Variables	Lowest tertile NLR <2.10 *N* = 35	Middle tertile NLR (2.10–3.82) *N* = 35	Highest tertile NLR (≥3.83) *N* = 36	*p*-value
Age (years), mean (SD)	64.7 (13.5)	63.5 (14.1)	64.9 (12.6)	0.898
Gender, male (%)	18 (51.4)	20 (57.1)	22 (61.1)	0.711
HTN, yes (%)	32 (91.4)	31 (88.6)	30 (83.3)	0.573
T2DM, yes (%)	12 (34.3)	6 (17.1)	10 (27.8)	0.260
WHR, mean (SD)	0.97 (0.11)	0.98 (0.13)	0.99 (0.12)	0.936
SBP, mmHg mean (SD)	153.5 (22.0)	161.5 (31.1)	155.9 (29.4)	0.472
DBP, mmHg, mean (SD)	91.4 (12.1)	92.9 (15.0)	94.8 (19.2)	0.650
PCV, mean (SD)	39.7 (4.6)	39.4 (5.3)	39.8 (6.1)	0.942
Serum RBG	121.4 (46.7)	141.9 (63.8)	151.0 (48.3)	0.170
Serum Cr, mean (SD)	1.07 (0.46)	1.35 (1.45)	1.38 (1.03)	0.41
Urea, mean (SD)	28.6 (12.7)	37.3 (23.2)	45.4 (26.5)	<0.007^*^
Serum RBG, mean (SD)	129.8 (53.9)	146.6 (54.9)	—	0.19
LDL, mean (SD)	138.2 (53.3)	131.0 (44.7)	129.5 (56.2)	0.806
HDL, mean (SD)	56.4 (21.8)	49.4 (15.7)	49.3 (14.0)	0.244
TC, mean (SD)	216.9 (65.8)	200.5 (55.4)	201.3 (67.4)	0.563
TG, mean (SD)	108.0 (35.5)	102.1 (39.4)	109.2 (45.4)	
WBC/ul, mean SD	6,712.0 (1,643.0)	7,421.1 (1,505.8)	8,975.3 (1,795.9)	<0.001^*^
Time from stroke onset to ED hours, median (IQR)	24.00 (55.00)	23.00 (32.00)	24.00 (31.00)	0.090
GCS, mean (SD)	14.8 (0.7)	14.2 (1.6)	12.1 (3.1)	<0.001^*^
NIHSS, mean (SD)	8.5 (3.2)	12.5 (3.3)	17.6 (4.6)	<0.001^*^
Hemisphere, left (%)	23 (65.7)	25 (71.4)	17 (47.2)	0.090
Location, subcortical (%)	27 (77.1)	15 (42.9)	6 (16.7)	0.001^*^
TOAST (%)				
Large vessel	1 (2.9)	3 (8.6)	8 (22.2)	<0.001^*^
Cardioembolic	0 (0)	6 (17.1)	12 (33.3)	
Small vessel	28 (80.0)	11 (31.4)	2 (5.6)	
Undetermined	6 (17.1)	14 (40.0)	14 (38.9)	
Determined	0 (0)	1 (2.9)	0 (0)	
Lesion size median (IQR)	1.50 (0.40)	3.80 (3.70)	5.70 (4.83)	<0.001^*^
Lesion volume median (IQR)	1.50 (0.86)	12.00 (21.65)	29.04 (25.80)	<0.001^*^
Duration of care, days mean (SD)	5.5 (2.4)	9.5 (5.8)	10.1 (6.5)	0.001^*^
Fever, yes (%)	0 (0)	16 (45.7)	31 (86.1)	<0.001^*^
Dysphagia, yes (%)	1 (2.9)	16 (45.7)	31 (88.6)	<0.001^*^
Dysphasia, yes (%)	7 (20.0)	15 (42.9)	17 (47.2)	0.039^*^
Aspiration pneumonia, yes (%)	0 (0)	3 (8.6)	25 (69.4)	<0.001^*^
30-day mRS, median (IQR)	2.0 (1.0)	3.0 (1.0)	5.0 (2.0)	<0.001^*^
30-day mRS poor outcome, 4–6 *N* (%)	4 (11.4)	14 (40.0)	29 (80.6)	<0.001^*^
30-day fatality *N* (%)	2 (5.7)	2 (5.7)	20 (18.9)	<0.001^*^

^*^p-value <0.05 was considered significant. HTN, hypertension; T2DM, type 2 diabetes mellitus; SBP, systolic blood pressure; DBP, diastolic blood pressure; GCS, Glasgow coma scale score; NIHSS, National Institutes of Health Stroke Scale; WHR, waist-hip ratio; PCV, packed cell volume; WBC, white blood cells; NLR, neutrophil-lymphocyte ratio; RBG, random blood glucose; LDL, low-density lipoprotein cholesterol; HDL, high-density lipoprotein cholesterol; TC, total cholesterol; TG, triglyceride; Cr, creatinine; ED, emergency department; UTI, urinary tract infection; DVT, deep vein thrombosis; mRS, modified Rankins scale score.

**Table 3:** Cox proportional regression showing the independent association between admitting NLR and poor 30-day outcome (L, left), and admitting NIHSS and 30-day outcome (R, right).

Co-variates	Adjusted hazards ratio and 95% confidence interval	Co-variates	Adjusted hazards ratio and 95% confidence interval
Admitting NLR	1.19 (1.01, 1.40)^**^	Admitting NIHSS	1.25 (1.14, 1.37)^**^
**Co-variates**		**Co-variates**	
Age	1.02 (0.99. 1.04)	Age	1.01 (0.99, 1.04)
Female gender	1.01 (0.54, 1.86)	Female gender	0.92 (0.49, 1.71)
Hypertension yes	1.47 (0.53, 4.06)	Hypertension yes	0.99 (0.35, 2.79)
T2DM, yes	0.82 (0.40, 1.67)	T2DM, yes	0.83 (0.39, 1.78)
Admitting GCS	0.91 (0.80, 1.03)	Admitting GCS	1.10 (0.93, 1.24)

^**^P value <0.05 was considered significant. T2DM, type 2 diabetes mellitus; GCS, Glasgow coma score.

## Discussion

In this study, using the ROC curve, we observed that the admitting NLR had similar diagnostic performance with the NIHSS score in discriminating patients with adverse 30-day functional outcome. Notably, the baseline NLR correlated strongly with the admitting NIHSS score, yielding a similar hazard ratio on the Cox proportional regression model. In many LMICs, where patients rarely have funds for endovascular intervention, admitting NLR, which requires little or no formal training, may help improve stroke triage decisions for intensive conservative care, which is often the only available care. Most stroke deaths occur within the first few weeks to months with the highest morbidity and mortality reported in many low and middle income countries (LMICs) (GBD 2021 Stroke Risk Factor Collaborators, 2024; Feigin et al., 2023; Owolabi et al., 2018). Paradoxically, however, the deaths and adverse outcomes attributable to stroke are largely preventable (Feigin et al., 2023) as they often result from an inevitable immune-inflammatory response following an acute cerebral ischemic event. With ongoing brain-drain in the health sector, many patients in West Africa, and indeed several other LMICs, present initially to non-neurologists, who find the deployment of the NIHSS score time-consuming in the acute care setting (Wahab, 2008). The patients also often present late and only a few ultimately benefit from interventional care (Adeoye et al., 2014). This makes protocols geared toward risk stratification highly desirable as conservative care is often the only available and affordable care.

Our study findings buttress the need for simplification of the acute stroke-care pathway, especially in resource-challenged settings (Feigin et al., 2023). The NIHSS score, which requires formal training for optimal application, is a documented predictor of outcome (Wouters et al., 2018), as is similarly shown in our study. However, in settings with few trained neurologists and specialist neurology paramedical experts, the NLR may be an equally useful tool. The NIHSS remains the standard requirement for patient inclusion in research and quality improvement initiatives; we therefore do not recommend disregarding the NIHSS from routine care assessments. However, in settings with increasing need for task shifting and task sharing, all healthcare providers and individuals must work together using readily available, routinely-obtained, and innovative low-cost strategies to reduce the burden of stroke and improve outcomes (Feigin et al., 2023). Given that the NLR is obtained as part of routine care, it brings no additional costs to the patient and no additional burden for training/certifications for allied health care providers. A tool like the NLR, in the right clinical setting, may help guide judicious allocation of scare resources (like stroke units, intensive care bedspaces, cardiac monitors) and also inform early referrals to specialist neuro-critical care centers.

Emerging experimental and clinical evidence suggest that brain–immune interactions play an important role in stroke outcome (Macrez et al., 2011; Hermann et al., 2018). Depending on the specific immune cells involved, these interactions may have protective, destructive, or regenerative effects on the brain (Dirnagl et al., 2007). While the benefit of NLR in predicting stroke outcome has been shown in several studies (Yu et al., 2018; Wang et al., 2019; Lux et al., 2020), mostly among the non-African ancestry population, no study has compared the benefit of NLR with the NIHSS in predicting stroke outcome. Our study provides evidence and helps bridge the research gap on the potential expanded utility of NLR in resource-challenged settings. Efforts targeted at surmounting the rising global burden of stroke must utilize solutions developed for and appropriate to LMICs (Owolabi et al., 2018).

In this study, we showed that admitting NLR was an independent predictor of adverse 30-day functional outcome with an HR of 1.19, having adjusted for age, gender, presence of hypertension, and T2DM as well admitting GCS score. It performed similarly to the admitting NIHSS with comparable hazard ratios (NLR: 1.19 vs. NIHSS: 1.25). At respective cut-off scores of 2.71 for the NLR and 12.5 for the NIHSS score, both parameters had similar sensitivities, while the latter had a higher specificity. This buttresses the usefulness of the NIHSS score in neurology practice. The comparative benefit of the NLR compared to the NIHSS is likely linked to the fact that patients with severe stroke, who tend to have worse outcomes, also have higher inflammatory burden and high risk of stroke-induced immunodepression (Dirnagl et al., 2007; Alsbrook et al., 2023; Amruta et al., 2020). They may then be at higher risk of post-stroke infections and other adverse complications (Amruta et al., 2020). A high NLR at baseline may similarly represent underlying prodromal stroke-associated infectious conditions, which invariably leads to worse outcomes. This is key in low-resource settings where post-stroke infections such as aspiration pneumonia and UTIs are still a common cause of mortality and adverse outcome in acute stroke settings (Sarfo et al., 2023). Patients with high NLR at admission may then be pre-emptively prioritized and targeted for early interventions, such as antimicrobial prophylaxis (Li et al., 2024; Vermeij et al., 2018; Rashid et al., 2020), the benefit of which may be better explored in future randomized controlled trials.

### Strengths and limitations

Although our patients were followed up during admission, serial measurement of the NLR over the time course of illness was not determined as this would be beyond the scope of the index study. While all participants were screened using stringent clinic–laboratory infection-exclusion parameters, subclinical infections may yet occur. In this study, data on long-term follow-up (beyond 30 days) were not readily available. However, in many LMICs, including Sub-Saharan Africa, stroke deaths and poor outcome typically occur within the 1^st^ week to the 1^st^ month (Komolafe et al., 2024; Akinyemi et al., 2021; Sarfo et al., 2023; Obiako et al., 2011; Ekeh et al., 2015). Our study therefore represents a notable and pioneering effort in prospectively evaluating the clinical utility of admitting NLR compared to the NIHSS score in identifying patients with poor stroke outcomes. Our findings are generalizable to many LMICs where there are few stroke-care professionals, and conservative care is often the only readily available and affordable care. This is especially important as the NLR could potentially help identify patients with adverse 30-day outcomes who may benefit from quick triaging, intensive conservative care, or urgent specialist referral.

## Conclusions and recommendations

Among Nigerian Africans, the admitting NLR, which gives an insight into the inflammatory burden of stroke, correlated strongly with and performed similarly to the NIHSS score and may help identify patients with adverse 30-day AIS outcome. In settings with limited capability for endovascular care and low neurologist-patient ratio, the admitting NLR may be a valuable initial tool for risk stratification and may aid acute care decisions, such as admission to stroke units, allocation of intensive care bedspaces, prophylactic use of antibiotics, and early neurology referral decisions. Stroke outcome prediction scores incorporating admitting NLR with other established markers may be beneficial amid efforts to improve risk stratification and optimize intensive conservative care of patients who are at high risk for adverse outcomes.

## Data Availability

The raw data supporting the conclusions of this article will be made available by the authors, without undue reservation.

## Footnotes

1 Za means Zα, the Z-score is approximately 1.96, when alpha (type 1 error) is = 0.05.

## References

[B1] Adebayo O. Akpa O. Asowata O. J. Fakunle A. Sarfo F. S. Akpalu A. . (2023). Determinants of first-ever stroke severity in West Africans: evidence from the SIREN study. J. Am. Heart Assoc. 12:e027888. doi: 10.1161/JAHA.122.027888 PMC10356032 37301737

[B2] Adeoye O. Albright K. C. Carr B. G. Wolff C. Mullen M. T. Abruzzo T. . (2014). Geographic access to acute stroke care in the United States. Stroke 45, 3019–3024. doi: 10.1161/STROKEAHA.114.006293 25158773 PMC5877807

[B3] Akinyemi R. O. Ovbiagele B. Adeniji O. A. Sarfo F. S. Abd-Allah F. Adoukonou T. . (2021). Stroke in Africa: profile, progress, prospects and priorities. Nat. Rev. Neurol. 17, 634–656. doi: 10.1038/s41582-021-00542-4 34526674 PMC8441961

[B4] Alsbrook D. L. Di Napoli M. Bhatia K. Biller J. Andalib S. Hinduja A. . (2023). Neuroinflammation in acute ischemic and hemorrhagic stroke. Curr. Neurol. Neurosci. Rep. 23, 407–431. doi: 10.1007/s11910-023-01282-2 37395873 PMC10544736

[B5] Amruta N. Rahman A. A. Pinteaux E. Bix G. (2020). Neuroinflammation and fibrosis in stroke: the good, the bad and the ugly. J. Neuroimmunol. 346:577318. doi: 10.1016/j.jneuroim.2020.577318 32682140 PMC7794086

[B6] Brott T. Adams H. P. Olinger C. P. Marler J. R. Barsan W. G. Biller J. . (1989). Measurements of acute cerebral infarction: a clinical examination scale. Stroke 20, 864–870. doi: 10.1161/01.STR.20.7.864 2749846

[B7] Charan J. Biswas T. (2013). How to calculate sample size for different study designs in medical research? Indian J. Psychol. Med. 35, 121–126. doi: 10.4103/0253-7176.116232 24049221 PMC3775042

[B8] De Haan R. Horn J. Limburg M. Van Der Meulen J. Bossuyt P. (1993). A comparison of five stroke scales with measures of disability, handicap, and quality of life. Stroke 24, 1178–1181. doi: 10.1161/01.STR.24.8.1178 8342193

[B9] Dewey H. M. Donnan G. A. Freeman E. J. Sharples C. M. Macdonell R. A. McNeil J. J. . (1999). Interrater reliability of the National Institutes of Health Stroke Scale: rating by neurologists and nurses in a community-based stroke incidence study. Cerebrovasc. Dis. 9, 323–327. doi: 10.1159/000016006 10545689

[B10] Dirnagl U. Klehmet J. Braun J. S. Harms H. Meisel C. Ziemssen T. . (2007). Stroke-induced immunodepression. Stroke 38, 770–773. doi: 10.1161/01.STR.0000251441.89665.bc 17261736

[B11] Ekeh B. Ogunniyi A. Isamade E. Ekrikpo U. (2015). Stroke mortality and its predictors in a Nigerian teaching hospital. Afr. Health Sci. 15, 74–81. doi: 10.4314/ahs.v15i1.10 25834533 PMC4370132

[B12] Elkind M. S. Cheng J. Boden-Albala B. Paik M. C. Sacco R. L. Northern Manhattan Stroke Study (2001). Elevated white blood cell count and carotid plaque thickness : the northern manhattan stroke study. Stroke 32, 842–849. doi: 10.1161/01.STR.32.4.842 11283380

[B13] Feigin V. L. Owolabi M. O. World Stroke Organization–Lancet Neurology Commission Stroke Collaboration Group (2023). Pragmatic solutions to reduce the global burden of stroke: a World Stroke Organization-Lancet Neurology Commission. Lancet Neurol. 22, 1160–1206. doi: 10.1016/S1474-4422(23)00277-6 37827183 PMC10715732

[B14] GBD 2021 Stroke Risk Factor Collaborators (2024). Global, regional, and national burden of stroke and its risk factors, 1990-2021: a systematic analysis for the Global Burden of Disease Study 2021. Lancet Neurol. 23, 973–1003. doi: 10.1016/S1474-4422(24)00369-7 39304265 PMC12254192

[B15] Goldstein L. B. Bertels C. Davis J. N. (1989). Interrater reliability of the NIH stroke scale. Arch. Neurol. 46, 660–662. doi: 10.1001/archneur.1989.00520420080026 2730378

[B16] Goldstein L. B. Samsa G. P. (1997). Reliability of the National Institutes of Health Stroke Scale. Extension to non-neurologists in the context of a clinical trial. Stroke 28, 307–310. doi: 10.1161/01.STR.28.2.307 9040680

[B17] Grau A. J. Boddy A. W. Dukovic D. A. Buggle F. Lichy C. Brandt T. . (2004). Leukocyte count as an independent predictor of recurrent ischemic events. Stroke 35, 1147–1152. doi: 10.1161/01.STR.0000124122.71702.64 15017013

[B18] Hermann D. M. Kleinschnitz C. Gunzer M. (2018). Implications of polymorphonuclear neutrophils for ischemic stroke and intracerebral hemorrhage: predictive value, pathophysiological consequences and utility as therapeutic target. J. Neuroimmunol. 321, 138–143. doi: 10.1016/j.jneuroim.2018.04.015 29729895

[B19] Kasner S. E. Chalela J. A. Luciano J. M. Cucchiara B. L. Raps E. C. McGarvey M. L. . (1999). Reliability and validity of estimating the NIH stroke scale score from medical records. Stroke 30, 1534–1537. doi: 10.1161/01.STR.30.8.1534 10436096

[B20] Kim J. Y. Park J. Chang J. Y. Kim S. H. Lee J. E. (2016). Inflammation after ischemic stroke: the role of leukocytes and glial cells. Exp. Neurobiol. 25, 241–251. doi: 10.5607/en.2016.25.5.241 27790058 PMC5081470

[B21] Komolafe M. A. Sunmonu T. Akinyemi J. Sarfo F. S. Akpalu A. Wahab K. . (2024). Clinical and neuroimaging factors associated with 30-day fatality among indigenous West Africans with spontaneous intracerebral hemorrhage. J. Neurol. Sci. 456:122848. doi: 10.1016/j.jns.2023.122848 38171072 PMC10888524

[B22] Kothari R. U. Brott T. Broderick J. P. Barsan W. G. Sauerbeck L. R. Zuccarello M. . (1996). The ABCs of measuring intracerebral hemorrhage volumes. Stroke 27, 1304–1305. doi: 10.1161/01.STR.27.8.1304 8711791

[B23] Li J. Meng X. Shi F. D. Jing J. Gu H. Q. Jin A. . (2024). Colchicine in patients with acute ischaemic stroke or transient ischaemic attack (CHANCE-3): multicentre, double blind, randomised, placebo controlled trial. BMJ 385:e079061. doi: 10.1136/bmj-2023-079061 38925803 PMC11200154

[B24] Lim E. M. Cembrowski G. Cembrowski M. Clarke G. (2010). Race-specific WBC and neutrophil count reference intervals. Int. J. Lab. Hematol. 32, 590–597. doi: 10.1111/j.1751-553X.2010.01223.x 20236184

[B25] Lux D. Alakbarzade V. Bridge L. Clark C. N. Clarke B. Zhang L. . (2020). The association of neutrophil-lymphocyte ratio and lymphocyte-monocyte ratio with 3-month clinical outcome after mechanical thrombectomy following stroke. J. Neuroinflam. 17:60. doi: 10.1186/s12974-020-01739-y PMC7026966 32070366

[B26] Lyden P. D. Lu M. Levine S. R. Brott T. G. Broderick J. NINDS rt PA Stroke Study Group (2001). A modified National Institutes of Health Stroke Scale for use in stroke clinical trials: preliminary reliability and validity. Stroke 32, 1310–1317. doi: 10.1161/01.STR.32.6.1310 11387492

[B27] Macrez R. Ali C. Toutirais O. Le Mauff B. Defer G. Dirnagl U. . (2011). Stroke and the immune system: from pathophysiology to new therapeutic strategies. Lancet Neurol. 10, 471–480. doi: 10.1016/S1474-4422(11)70066-7 21511199

[B28] Makharia A. Agarwal A. Garg D. Vishnu V. Y. Srivastava M. V. P. (2024). The pitfalls of NIHSS: time for a new clinical acute stroke severity scoring system in the emergency? Ann. Indian Acad. Neurol. 27:15. doi: 10.4103/aian.aian_842_23 38495237 PMC10941908

[B29] Martin-Schild S. Albright K. C. Tanksley J. Pandav V. Jones E. B. Grotta J. C. . (2011). Zero on the NIHSS does not equal the absence of *Stroke Ann. Emerg. Med*. 57, 42–45. doi: 10.1016/j.annemergmed.2010.06.564 PMC3426834 20828876

[B30] Meisel C. Schwab J. M. Prass K. Meisel A. Dirnagl U. (2005). Central nervous system injury-induced immune deficiency syndrome. Nat. Rev. Neurosci. 6, 775–786. doi: 10.1038/nrn1765 16163382

[B31] Meyer B. C. Hemmen T. M. Jackson C. M. Lyden P. D. (2002). Modified National Institutes of Health Stroke Scale for use in stroke clinical trials: prospective reliability and validity. Stroke 33, 1261–1266. doi: 10.1161/01.STR.0000015625.87603.A7 11988601

[B32] Meyer B. C. Lyden P. D. (2009). The modified National Institutes of Health Stroke Scale: its time has come. Int. J. Stroke 4, 267–273. doi: 10.1111/j.1747-4949.2009.00294.x 19689755 PMC2729912

[B33] Nam K. W. Kim T. J. Lee J. S. Kwon H. M. Lee Y. S. Ko S. B. . (2018). High neutrophil-to-lymphocyte ratio predicts stroke-associated pneumonia. Stroke 49, 1886–1892. doi: 10.1161/STROKEAHA.118.021228 29967014

[B34] Ni A. (2016). Reference values of neutrophil-lymphocyte ratio, platelet-lymphocyte ratio and mean platelet volume in healthy adults in North Central Nigeria. J. Blood Lymph 6:68492. doi: 10.4172/2165-7831.1000143

[B35] Obiako O. Ogunniyi A. Oparah S. (2011). Prognosis and outcome of acute stroke in the University College Hospital Ibadan, Nigeria. Niger. J. Clin. Pract. 14:359. doi: 10.4103/1119-3077.86784 22037085

[B36] Offner H. Subramanian S. Parker S. M. Wang C. Afentoulis M. E. Lewis A. . (2006). Splenic atrophy in experimental stroke is accompanied by increased regulatory T cells and circulating macrophages. J. Immunol. 176, 6523–6531. doi: 10.4049/jimmunol.176.11.6523 16709809

[B37] Ojagbemi A. Owolabi M. Atalabi M. Baiyewu O. (2013). Stroke lesions and post-stroke depression among survivors in Ibadan, Nigeria. Afr. J. Med. Med. Sci. 42, 245–251. 24579386

[B38] Omuse G. Maina D. Mwangi J. Wambua C. Radia K. Kanyua A. . (2018). Complete blood count reference intervals from a healthy adult urban population in Kenya. PLoS ONE 13:e0198444. doi: 10.1371/journal.pone.0198444 29879171 PMC5991659

[B39] Owolabi M. Johnson W. Khan T. Feigin V. (2018). Effectively combating stroke in low- and middle-income countries: placing proof in pragmatism—the lancet neurology commission. J. Stroke Med. 1, 65–67. doi: 10.1177/2516608518776706

[B40] Owolabi M. O. Platz T. (2008). Proposing the stroke levity scale: a valid, reliable, simple, and time-saving measure of stroke severity. Eur. J. Neurol. 15, 627–633. doi: 10.1111/j.1468-1331.2008.02140.x 18474078

[B41] Rashid M. H. Kabir A. Waris M. U. Salman U. Zain S. (2020). Role of prophylactic antibiotics in critical care of stroke patients - a preventive approach to post-stroke infections? Cureus 12:e7158. doi: 10.7759/cureus.7158 32257701 PMC7108674

[B42] Reed W. W. Diehl L. F. (1991). Leukopenia, neutropenia, and reduced hemoglobin levels in healthy American blacks. Arch. Intern. Med. 151, 501–505. doi: 10.1001/archinte.1991.00400030063011 2001132

[B43] Reich D. Nalls M. A. Kao W. H. L. Akylbekova E. L. Tandon A. Patterson N. . (2009). Reduced neutrophil count in people of african descent is due to a regulatory variant in the duffy antigen receptor for chemokines gene. PLoS Genet. 5:e1000360. doi: 10.1371/journal.pgen.1000360 19180233 PMC2628742

[B44] Sacco R. L. Kasner S. E. Broderick J. P. Caplan L. R. Connors J. J. Culebras A. . (2013). An updated definition of stroke for the 21st Century. Stroke 44, 2064–2089. doi: 10.1161/STR.0b013e318296aeca 23652265 PMC11078537

[B45] Sarfo F. S. Akpa O. M. Ovbiagele B. Akpalu A. Wahab K. Obiako R. . (2023). Patient-level and system-level determinants of stroke fatality across 16 large hospitals in Ghana and Nigeria: a prospective cohort study. Lancet Glob Health. 11, e575–e585. doi: 10.1016/S2214-109X(23)00038-4 36805867 PMC10080070

[B46] Schmülling S. Grond M. Rudolf J. Kiencke P. (1998). Training as a prerequisite for reliable use of NIH Stroke Scale. Stroke 29, 1258–1259. doi: 10.1161/01.STR.29.6.1258 9626306

[B47] Thobakgale C. F. (2014). Ndung'u T. Neutrophil counts in persons of African origin. Curr. Opin. Hematol. 21, 50–57. doi: 10.1097/MOH.0000000000000007 24257098

[B48] Tirschwell D. L. Longstreth W. T. Becker K. J. Gammans R. E. Sabounjian L. A. Hamilton S. . (2002). Shortening the NIH stroke scale for use in the prehospital setting. Stroke 33, 2801–2806. doi: 10.1161/01.STR.0000044166.28481.BC 12468773

[B49] Vermeij J. D. Westendorp W. F. van de Beek D. Nederkoorn P. J. (2018). Post-stroke infections and preventive antibiotics in stroke: update of clinical evidence. Int. J. Stroke 13, 913–920. doi: 10.1177/1747493018798557 30175940

[B50] Wahab K. W. (2008). The burden of stroke in Nigeria. Int. J. Stroke 3, 290–292. doi: 10.1111/j.1747-4949.2008.00217.x 18811746

[B51] Wang L. Song Q. Wang C. Wu S. Deng L. Li Y. . (2019). Neutrophil to lymphocyte ratio predicts poor outcomes after acute ischemic stroke: a cohort study and systematic review. J. Neurol. Sci. 406:116445. doi: 10.1016/j.jns.2019.116445 31521961

[B52] Woo D. Broderick J. P. Kothari R. U. Lu M. Brott T. Lyden P. D. . (1999). Does the National Institutes of Health Stroke Scale favor left hemisphere strokes? NINDS t-PA stroke study group. Stroke 30, 2355–2359. doi: 10.1161/01.STR.30.11.2355 10548670

[B53] Wouters A. Nysten C. Thijs V. Lemmens R. (2018). Prediction of outcome in patients with acute ischemic stroke based on initial severity and improvement in the first 24 h. Front. Neurol. 9:308. doi: 10.3389/fneur.2018.00308 29867722 PMC5950843

[B54] Yu S. Arima H. Bertmar C. Clarke S. Herkes G. Krause M. . (2018). Neutrophil to lymphocyte ratio and early clinical outcomes in patients with acute ischemic *Stroke J. Neurol. Sci*. 387, 115–118. doi: 10.1016/j.jns.2018.02.002 29571846

[B55] Zhao L. Dai Q. Chen X. Li S. Shi R. Yu S. . (2016). Neutrophil-to-lymphocyte ratio predicts length of stay and acute hospital cost in patients with acute ischemic stroke. J. Stroke Cerebrov. Dis. 25, 739–744. doi: 10.1016/j.jstrokecerebrovasdis.2015.11.012 26775271

