# Correction: Comparison of admitting neutrophil/lymphocyte ratio with baseline NIH stroke scale score in discriminating poor 30-day stroke outcome among Nigerian Africans

**DOI:** 10.3389/fstro.2026.1917786

**Published:** 2026-07-06

**Authors:** Oladotun V. Olalusi, Joseph Yaria, Akintomiwa Makanjuola, Rufus Akinyemi, Mayowa Owolabi, Adesola Ogunniyi

**Affiliations:** 1 Department of Neurology, University College Hospital, Ibadan, Nigeria; 2 Neuroscience and Aging Research Unit, Institute of Advanced Medical Research and Training, College of Medicine, University of Ibadan, Ibadan, Nigeria; 3 Center for Genomics and Precision Medicine, College of Medicine, University of Ibadan, Ibadan, Nigeria; 4 College of Medicine, University of Ibadan, Ibadan, Nigeria; 5 Department of Medicine, Lebanese American University of Beirut, Beirut, Lebanon; 6 Department of Medicine, Blossom Specialist Medical Center, Ibadan, Nigeria

**Keywords:** neutrophil-lymphocyte ratio (NLR), NIH Stroke Scale (NIHSS) score, modified Rankins scale (mRS) score, acute ischemic stroke (AIS), 30-day functional outcome, ischemic stroke mortality, Nigerian Africans, Indigenous West Africans

The funder [National Institutes of Health], [SIREN (U54HG007479), Systematic Investigation of Blacks with Stroke (SIBS) Genomics (R01NS107900), SIBS Genetics Genomics Counselling (SIBS Gen Gen) (R01NS107900-02S1), African Rigorous Innovative Stroke Epidemiological Surveillance (ARISES) (R01NS115944-01), H3Africa CVD Supplement (3U24HG009780-03S5), CNV in Stroke (CaNVAS) (1R01NS114045–01), Sub-Saharan Africa Conference on Stroke (SSACS) 1R13NS115395–01A1, Training Africans to Lead and Execute Neurological Trials & Studies (TALENTS) (D43TW012030) and Growing Data-science Research in Africa to Stimulate Progress (GRASP) (1UE5HL172183–01)] to [Mayowa Owolabi] was erroneously omitted.

**Acknowledgments**

In the **Acknowledgments**, [The authors thank the Departments of Medicine and Neurology, University College Hospital Ibadan, Nigeria, the Stroke and Cardiovascular Research Training (S-CaRT) institute, and the West African College of Physicians, specialty] was erroneously published. It now reads: [The authors thank the Departments of Medicine and Neurology, University College Hospital Ibadan, Nigeria, the Stroke and Cardiovascular Research Training (S-CaRT) institute, and the West African College of Physicians, Neurology specialty as well as the Training Africans to Lead and Execute Neurological Trials and Studies (TALENTS) program.]

The original version of this article has been updated.

