# Supplementary material for: Comparison of admitting neutrophil/lymphocyte ratio with baseline NIH stroke scale score in discriminating poor 30-day stroke outcome among Nigerian Africans
Source: Front Stroke. 2025 Apr 9;4:1562048. doi: 10.3389/fstro.2025.1562048 (PMC12802611; doi:10.3389/fstro.2025.1562048)
Supplement: Supplementary file 1 [file Data_Sheet_1.docx]

**COMPARISON OF ADMITTING NEUTROPHIL/LYMPHOCYTE RATIO WITH BASELINE NIH STROKE SCALE SCORE IN DISCRIMINATING POOR 30-DAY STROKE OUTCOME AMONG NIGERIAN AFRICANS**

Oladotun V. Olalusi^1,2^*, Joseph Yaria^1^, Akintomiwa Makanjuola^1^, Rufus Akinyemi^1,2,4^, Mayowa Owolabi^1,3,4,5,6^^, Adesola Ogunniyi^1,3^^

^1^ Department of Neurology, University College Hospital, Ibadan Nigeria

^2^ Neuroscience and aging research unit, Institute of Advanced Medical Research and Training, College of Medicine, University of Ibadan, Nigeria

^3^ College of Medicine, University of Ibadan, Nigeria

^4^ Center for Genomics and Precision Medicine, College of Medicine, University of Ibadan, Nigeria

^5^ Lebanese American University of Beirut

^6^ Blossom Specialist Medical Center, Ibadan, Nigeria

^ Senior authors

* Correspondence:

Dr Oladotun V. Olalusi, MD, Certificate Clinical Research (South Carolina), FWACP (Neurol)

Department of Neurology, University College Hospital, Ibadan, Nigeria

Tel: +234 703 806 5970 Email: [oladotunvolalusi@gmail.com](mailto:oladotunvolalusi@gmail.com)

**Keywords:** Neutrophil-Lymphocyte Ratio, NLR, Acute ischemic stroke, 30-day functional outcome, ischemic stroke mortality, West Africans

**List of tables and figures**: Tables 1-3, figures 1

Supplements: Tables S1, Figure S1

**Supplementary Table S1:**

|  | CENTERS FOR DISEASE CONTROL AND PREVENTION/NATIONAL HEALTHCARE SAFETY NETWORK (CDC/NHSN) surveillance definition of healthcare-associated infection and criteria for specific types of infections in the acute care setting |
| --- | --- |
| UTI-URINARY TRACT INFECTION SUTI-Symptomatic urinary tract infection | Asymptomatic urinary tract infection must meet at least 1 of the following criteria:  1. Patient has at least 1 of the following signs or symptoms with no other recognized cause: fever (>38C), urgency, frequency, dysuria, or suprapubic tenderness  The patient has a positive urine culture, that is, ≥105 microorganisms per cc of urine with no more than 2 species of microorganisms.  2. Patient has at least 2 of the following signs or symptoms with no other recognized cause: fever (>38C), urgency, frequency, dysuria, or suprapubic tenderness, and at least 1 of the following  a. positive dipstick for leukocyte esterase and/ or nitrate  b. pyuria (urine specimen with ≥10 white blood cells [WBC]/mm3 or ≥3 WBC/high-power field of unspun urine)  c. organisms seen on Gram’s stain of unspun urine  d. at least 2 urine cultures with repeated isolation of the same uro-pathogen (gram-negative bacteria or Staphylococcus saprophyticus) with ≥102 colonies/mL in non-voided specimens  e. ≤105 colonies/mL of a single uro-pathogen (gram-negative bacteria or S saprophyticus) in a patient being treated with an effective antimicrobial agent for a urinary tract infection  f. physician diagnosis of a urinary tract infection  g. physician institutes appropriate therapy for a urinary tract infection. |
| CLINICALLY-DEFINED PNEUMONIA | Symptoms and signs  FOR ANY PATIENT, at least 1 of the following:  Fever (>38C) with no other recognized cause  Leukopenia (<4000 WBC/mm3) or leukocytosis (>12,000 WBC/mm3 )  For adults >70 years old, altered mental status with no other recognized cause  and at least 2 of the following:  New onset of purulent sputum or change in character of sputum or increased respiratory secretions or increased suctioning requirements  New onset or worsening cough, or dyspnea, or tachypnea  Rales or bronchial breath sounds  Worsening gas exchange (e.g., O2 desaturations [e.g., PaO2/FiO2 <240], increased oxygen requirements, or increased ventilator demand)  RADIOLOGY  Two or more serial chest radiographs with at least 1 of the following: New or progressive and persistent infiltrate, Consolidation, Cavitation, Pneumatoceles, in infants <1 year old  NOTE: In patients without underlying pulmonary or cardiac disease (e.g., respiratory distress syndrome, bronchopulmonary dysplasia, pulmonary edema, or chronic obstructive pulmonary disease), 1 definitive chest radiograph is acceptable. |
| GE-Gastroenteritis | Gastroenteritis must meet at least 1 of the following criteria:  1. Patient has an acute onset of diarrhea (liquid stools for more than 12 hours) with or without vomiting or fever (.388C) and no likely noninfectious cause (e.g., diagnostic tests, therapeutic regimen other than antimicrobial agents, acute exacerbation of a chronic condition, or psycho- logic stress).  2. Patient has at least 2 of the following signs or symptoms with no other recognized cause: nausea, vomiting, abdominal pain, fever (.388C), or headache and at least 1 of the following:  a) an enteric pathogen is cultured from stool or rectal swab  b) an enteric pathogen is detected by routine or electron microscopy  c) an enteric pathogen is detected by antigen or antibody assay on blood or feces  d) evidence of an enteric pathogen is detected by cytopathic changes in tissue culture (toxin assay)  e) diagnostic single antibody titer (IgM) or 4-fold increase in paired sera (IgG) for the pathogen. |
| SST-SKIN AND SOFT TISSUE INFECTION SKIN-Skin | Skin infections must meet at least 1 of the following criteria:  1. Patient has purulent drainage, pustules, vesicles, or boils.  2. Patient has at least 2 of the following signs or symptoms with no other recognized cause: pain or tenderness, localized swelling, redness, or heat and at least 1 of the following:  a. organisms cultured from aspirate or drain age from the affected site; if organisms are normal skin flora (i.e., diphtheroids [Corynebacterium spp.], Bacillus [not B anthracis] spp., Propionibacterium spp., coagulase-negative staphylococci [including S epidermidis], viridans group streptococci, Aerococcus spp., Micrococcus spp.), they must be a pure culture  b. organisms cultured from blood  c. positive antigen test performed on infected tissue or blood (e.g., herpes simplex, varicella zoster, H influenzae, N meningitidis)  d. multinucleated giant cells seen on microscopic examination of affected tissue  e. diagnostic single antibody titer (IgM) or 4-­fold increase in paired sera (IgG) for  pathogen. |

**SUPPLEMENTAL FILE:**


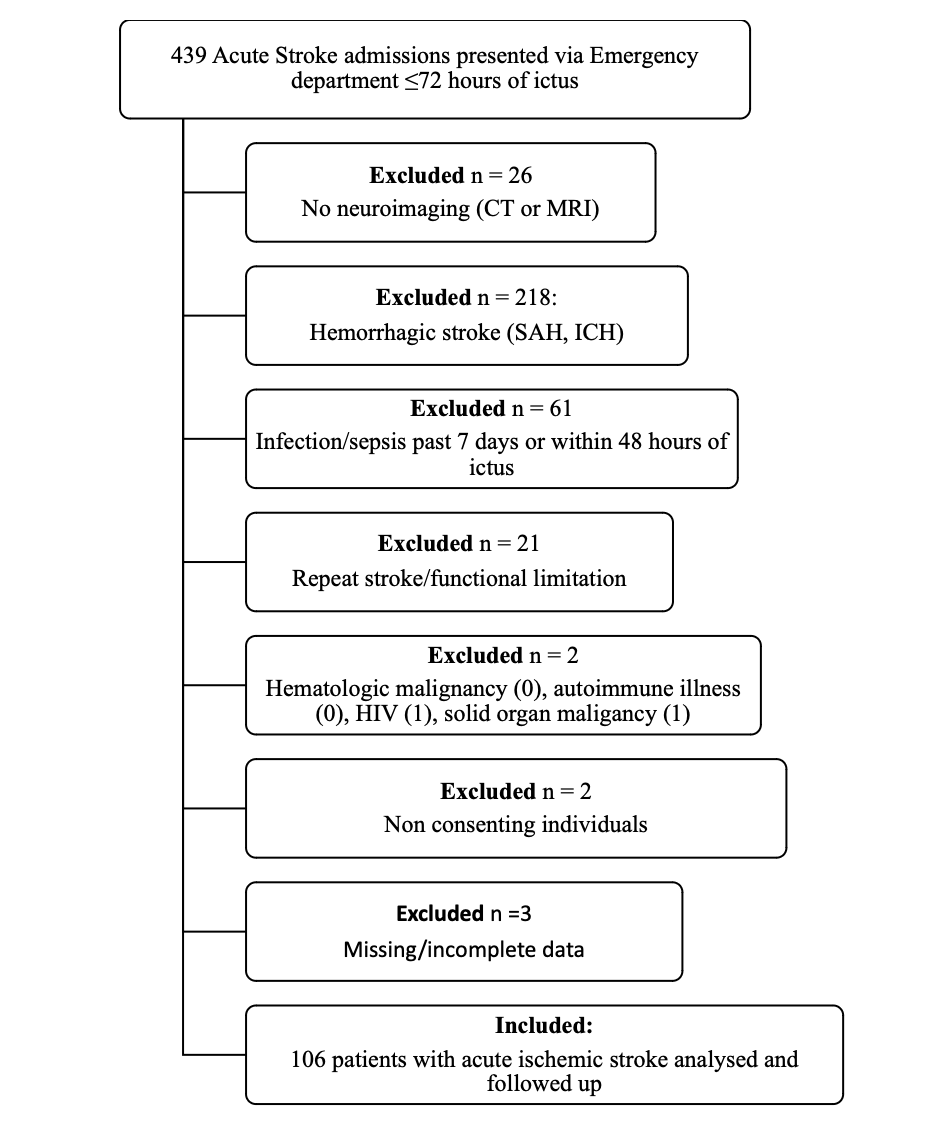


Stroke participants’ recruitment flowchart (supplementary figure S1)

CT: computed tomography, MRI: magnetic resonance imaging, SAH, sub-arachnoid hemorrhage, ICH, intracerebral hemorrhage; HIV, human immunodeficiency virus

Patients were excluded if they had 1) clinico-laboratory evidence of infection/sepsis at admission (which may cause admitting neutrophilia and thus confound the baseline NLR), 2) hemorrhagic stroke (a more severe stroke subtype with a higher risk of infectious complications at admission and fewer benefits from immunomodulatory/anti-inflammatory therapy), 3) previous stroke (associated with pre-stroke functional disability, thus confounding the mRS score), 4) underlying chronic immune/inflammatory disease, hematologic malignancy, or use of NSAIDS/steroids or immune modulatory agents.
